# A Highly Thermostable Xylanase from *Stenotrophomonas maltophilia*: Purification and Partial Characterization

**DOI:** 10.1155/2013/429305

**Published:** 2013-12-14

**Authors:** Abhay Raj, Sharad Kumar, Sudheer Kumar Singh

**Affiliations:** ^1^Environmental Microbiology Section, CSIR-Indian Institute of Toxicology Research (IITR), M. G. Marg, P.O. Box No. 80, Lucknow 226 001, India; ^2^Microbiology Division, CSIR-Central Drug Research Institute (CDRI), Sector 10, Jankipuram Extension, Sitapur Road, Lucknow 226 031, India

## Abstract

Seven xylanolytic bacterial strains were isolated from saw-dust dump soil. The bacterial strain X6 was selected on the basis of the highest xylanase activity with no cellulase contamination. It was identified as *Stenotrophomonas maltophilia* by biochemical tests and 16S rRNA gene sequencing approach. Xylanase production studies by *S. maltophilia* on different commercial xylans and agro-industrial residues suggested that wheat bran was the best carbon source for xylanase production (26.4 ± 0.6 IU/mL). The studies with inorganic and organic nitrogen sources suggested yeast extract as the best support for xylanase production (25 ± 0.6 IU/mL). Maximum xylanase production was observed at initial medium pH = 8.0 (23.8 ± 0.4 IU/mL) with production at pH = 7.0 and pH = 9.0 being almost comparable. Xylanase produced by *S. maltophilia* was purified to homogeneity using ammonium sulfate precipitation, gel filtration, and ion exchange chromatography. The final purification was 5.43-fold with recovery of 19.18%. The molecular weight of the purified xylanase protein was ~142 kDa. Both crude and purified xylanase had good stability at pH = 9.0 and 80°C with activity retention greater than 90% after 30 min incubation. The enzyme stability at high temperature and alkaline pH make it potentially effective for industrial applications.

## 1. Introduction

Xylan is a biopolymer comprising of D-xylose monomers which are linked through *β*-1,4-glycosyl bond, and is found abundantly in lignocellulosic biomass. Due to complex structure of xylan, many different enzymes are needed for its complete degradation, but xylanase (EC 3.2.1.8) is sufficient to break down the xylan backbone. Xylanases are produced by different species of microorganisms and have been studied mostly from bacteria, actinomycetes, and fungi [1–3]. The xylanolytic enzymes of microbial origin are of great significance for biotechnological applications in various industries such as animal feed preparation, food processing, textiles, pharmacy, paper, and pulp industries [4]. The xylanases have immense potential for production of several valuable products like xylitol and ethanol at low cost [5]. One of the potential uses of xylanase is the development of eco-friendly biobleaching method used in paper industry, where it is used as pretreatment prior to bleaching of kraft-cooked pulp to reduce application of chlorine and toxicity of chlorine bleached effluent [6]. The pulp bleaching process requires xylanases which are thermostable (>70°C) and active in alkaline condition (>8.0 = pH), due to alkaline and hot nature of the kraft-cooked pulp [6].

The xylanases have been isolated and characterized from different sources and evaluated for pulp bleaching properties [3, 7]. Most of the xylanases characterized so far showed optimum activity at acidic, neutral, or slightly alkaline pH (7.0-8.0) and at temperatures between 40°C and 60°C. Also, despite extensive exploitation of microbial diversity for novel xylanase producers, however, xylanases that are thermostable and alkali tolerant are limited [2, 8]. In the present study, xylanolytic *Stenotrophomonas maltophilia* was isolated from saw-dust dump and cultural conditions were studied for maximum xylanase production. Further, the enzyme produced was subjected to purification and characterization.

## 2. Materials and Methods

### 2.1. Chemicals, Media, and Media Components

Xylans, 3,5-dinitrosalicylic acid (DNSA), carboxymethyl cellulose (CMC), Sephadex G-100, DEAE-Cellulose, Congo red, and D-xylose were purchased from Sigma (St. Louis, MO, USA). Bacteriological peptone, yeast extract, tryptone, beef extract, D-glucose, nutrient agar, nutrient broth, and agar powder were obtained from HiMedia Laboratories Pvt., Ltd., Mumbai, India. All other chemicals used in this work were of analytical grade and obtained from S. D. Fine Chem. Ltd., Mumbai, India and SRL Chemicals, India. Wheat bran and rice bran were obtained from local market.

### 2.2. Isolation of Xylanolytic Bacteria

The xylanolytic bacteria were isolated from soil sample collected from a saw-dust dump located around a saw mill in Lucknow, India. The isolation procedure was as provided: 1 g soil was suspended in 9 mL sterile normal saline, agitated for one min, and 0.1 mL suspension was spread over xylan agar (pH = 8.0) plate and incubated at 37°C for 48 hours. The xylan agar contained (g/L): NaNO_3_ 3.0, K_2_HPO_4_ 0.5, MgSO_4_·7H_2_O 0.2, MnSO_4_·H_2_O 0.02, FeSO_4_·H_2_O 0.02, and CaCl_2_·2H_2_O 0.02 [9], yeast extract 2.0, agar powder 16.0, and birchwood xylan 10.0 as a source of carbon. The plates were incubated at 37°C for 48 hours and colonies with distinct morphologies were picked up and restreaked on nutrient agar (g/L): peptic digest of animal tissue 5.0, sodium chloride 5.0, beef extract 1.5, yeast extract 1.5, and agar powder 15.0. The xylanolytic activity of bacterial isolates was further confirmed by growing individual isolate on xylan agar plate at 37°C for 48 hours. The plates were stained with Congo red solution (0.1%, w/v) for 15 min and washed with (1 M) NaCl solution [10]. The xylanase-positive isolates, as determined by zone of clearance around the colony, were selected and maintained on xylan agar slant at 4°C.

### 2.3. Quantitative Assay

The xylanase-positive isolates were further studied for xylanase production using xylan production medium (xylan agar medium devoid of agar powder). The 1.0 mL of overnight grown culture in nutrient broth with an absorbance of 0.5 OD (A600; 1 cm cuvette) was used to inoculate 99 mL of xylanase production medium (pH = 8.0) in 250 mL Erlenmeyer flasks and incubated at 35°C, 120 rpm for 72 h (Innova, New Brunswick, USA). The broth was periodically withdrawn after 24 hours interval and centrifuged at 10000 ×g for 10 min (Sigma 3K-30, UK). The culture supernatant was used for xylanase assay [11]. The details of xylanase assay are as provided: the reaction mixture, consisting of 0.5 mL of culture supernatant and 1.5 mL of 1.0% birchwood xylan in 0.1 M phosphate buffer (pH = 8.0), was incubated at 50°C for 15 min. The reaction was terminated by adding 2.0 mL of 3,5-dinitrosalicylic acid (DNSA) reagent and boiling at 100°C for 10 min. The color formation was monitored at 540 nm and quantified by comparison with a standard curve of D-xylose. One unit of xylanase activity (IU) was defined as the amount of enzyme releasing 1 *μ*M of reducing sugars equivalent to D-xylose per minute under assay conditions. Cellulase activity was assayed by the same method, except low viscosity carboxymethyl cellulose (CMC) was used as substrate. One unit of cellulase activity was defined as the amount of enzyme releasing 1 *μ*M reducing sugar equivalent to glucose per minute under assay conditions. The highest xylanase producing isolate was selected for further studies.

### 2.4. Determination of Inducible/Constitutive Nature

The inducible/constitutive nature of xylanase was studied by performing production in medium differing by the presence of xylan/glucose (0.5% w/v) as carbon source. The production study was performed at 35°C, 120 rpm for 48 hours. The culture broth was centrifuged for 10 min at 10000 ×g and the supernatant was collected for xylanase activity detection on xylan agar plates. Xylan agar plate was prepared by dissolving birchwood xylan (0.45% w/v) and agar (1.8% w/v) in 0.1 M phosphate buffer (pH = 7.0). The 50 *μ*L of culture supernatant was loaded in the wells (diameter, 5 mm) made in plate and incubated overnight at 37°C in an upright position. The plate was observed for zone of clearance around the wells without Congo red staining.

### 2.5. Characterization of Xylanase Producer

The highest xylanase producer was preliminarly characterized by morphological and biochemical tests as per standard method [12]. This was further confirmed by 16S rRNA sequence analysis. The details are briefly described: total genomic DNA was extracted and purified from the strain using UltraClean microbial DNA isolation kit (MO BIO Lab Inc., USA). PCR amplification was performed with 16S rRNA universal primers: 27F (5′-AGAGTTTGATCCTGGCTCAG-3′) and 1492R (5′-TACGGTTACCTTGTTACGACTT-3′). The DNA fragment of ~1.45 kb was cut and eluted (Qiagen gel extraction kit). The sequencing was performed using the ABI 3130 genetic analyzer with Big Dye Terminator version 3.1 cycle sequencing kit. The sequence data was deposited in NCBI (National Centre for Biotechnology Information, http://www.ncbi.nlm.nih.gov/) GenBank and also used for BLAST (Basic Local Alignment Search Tool) analysis.

### 2.6. Effect of Culture Conditions on Production

Various cultural, nutritional, and physical parameters such as inoculum size, carbon and nitrogen source, and pH were studied to assess their effect on xylanase production. The production studies were performed at 35°C, 120 rpm for 48 hours. To study the effect of inoculum size, xylanase production medium in flasks were inoculated with different amounts (1.0%, 2.5%, 5.0% and 10% v/v) of 24 hours old culture. The effect of carbon source on xylanase production was studied by substituting birchwood xylan from xylanase production medium with beechwood xylan or with agroindustrial residues such as wheat bran and rice bran (1.0% w/v) as sole source of carbon. The flask with birchwood xylan was taken as control. Similarly, to study the effect of additional nitrogen supplementation, yeast extract was replaced with different inorganic (urea, ammonium sulphate, ammonium nitrate, sodium nitrate, or potassium nitrate) and organic (peptone, tryptone, or beef extract) nitrogen source (0.5%, w/v). The flask with yeast extract was used as control. The effect of pH on xylanase production was studied by adjusting the initial pH of xylanase production medium from pH = 6.0–10.0. The pH adjustment was performed using HCl (1 N) and Na_2_CO_3_ (2.0% w/v). The flask with pH = 8.0 was used as a control.

### 2.7. Purification of Extracellular Xylanase

The isolate X6 was cultivated in xylanase production medium containing wheat bran at 35°C, pH = 8.0 for 48 hours. The culture broth was centrifuged at 10,000 ×g for 20 min and filtered through 0.45 *μ*m filters (Millex Durapore, Millipore) to remove bacterial cells. The crude xylanase was brought from 0%–80% ammonium sulfate saturation with constant stirring and kept refrigerated for 2 hours. Afterwards, it was centrifuged and pellet was dissolved in 0.05 M sodium phosphate buffer (pH = 7.0). The protein was dialysed at 4°C for overnight against the same buffer using 12 kDa cut-off membrane (Himedia, LA395-5MT). The 20 mL of dialyzed preparation was loaded on gel filtration column (10 mm × 30 cm) packed with Sephadex G-100 previously equilibrated with 0.05 M sodium phosphate buffer (pH = 7.0) containing 50 mM NaCl. Elution of the protein was carried out with the same buffer at a flow rate of 18 mL/hour. Each fraction was analyzed for protein and xylanase activity. The xylanase activity fractions were pooled, desalted, and concentrated by ultrafiltration using Amicon Ultra-15 10 kDa (Millipore). Concentrated enzyme (2 mL) was applied on ion-exchange column (1.5 cm × 30 cm) packed with DEAE-cellulose that had been preequilibrated with 0.05 M sodium phosphate buffer (pH = 7.0). The column was first eluted with 40 mL 0.05 M sodium phosphate buffer to remove the unbound proteins and then with a 0.1 to 0.6 M NaCl gradient at a flow rate of 30 mL/hour. All the steps were carried out at 4°C to 8°C. The protein concentration in chromatographic eluates was determined by measuring the absorbance at 280 nm. Total protein content at the end of each purification step was quantified by the Bradford method [13], using the Genei protein estimation kit (Bangalore Genei, India).

### 2.8. SDS-PAGE and Zymogram Analysis

The SDS-PAGE analysis of purified xylanase was performed using 10% SDS-PAGE gel [14]. Electrophoresis was carried out using Mini-Gel Electrophoresis unit (Microkin, Techno Source, Mumbai, India) and proteins bands were visualized by Coomassie brilliant blue R-250 staining. The molecular weights of proteins were estimated using prestained protein ladder of 10–175 kDa range (NEX-GEN Pink-ADD; Puregene, Genetix Brand). Native-PAGE of the samples was carried out to study the zymogram. The procedure followed was similar to SDS-PAGE described above, but reducing agents such as SDS and mercaptoethanol were not added in the buffer/tracking dye solution. The step of samples boiling was eliminated during protein sample preparation. The samples were loaded in duplicate. After completion of the electrophoresis, the gel was cut into two parts, each with one set of samples. One set was stained with Coomassie brilliant blue R-250 staining to locate the position of protein and the other portion was used for zymogram analysis. For zymogram analysis, the gel was flooded with 1.0% (w/v) Birchwood xylan solution (prepared in 0.05 M phosphate buffer, pH = 7.0) and incubated at 37°C for 30 min. The gel was stained with Congo red solution (0.1%, w/v) for 15 min at room temperature and destained with NaCl (1 M) solution to visualize the clearing zone of hydrolysis.

### 2.9. Determination of Exo/Endoxylanolytic Activity

The exo/endoxylanolytic nature of the *S. maltophilia* xylanase was studied by carrying out thin layer chromatography (TLC) on silica gel plate. The standard xylan, xylanase treated xylan (1 mL of 1.0% xylan incubated with 50 *μ*L of purified xylanase for 1 hour at 37°C) were applied on plate and TLC was run using acetone-ethyl acetate-acetic acid (2 : 1 : 1 v/v/v) solvent system. The plate was developed by spraying 99.5% ethanol-sulfuric acid mix (95 : 5 v/v) and visualized after heating at 100°C until black spots became visible. D-Xylose (5 mg/mL) was used as a standard.

### 2.10. Determination of pH and Thermal Stability

The effect of pH on stability of crude and purified xylanase was studied by preincubating into 0.1 M of citrate buffer (pH = 4.0–pH = 6.0), phosphate buffer (pH = 6.0–pH = 8.0), Tris-HCl buffer (pH = 8.0–pH = 9.0), and glycine-NaOH buffer (pH = 9.0–pH = 11.0) at 50°C for 30 min and residual xylanase activity was measured by the method described earlier. The incubation at pH = 9.0 was treated as control. To determine the temperature stability, the crude and purified xylanase was preincubated for 30 min at different temperatures (30°C–100°C). The incubation at 50°C was used as control. The treated enzyme was transferred to 4°C and kept for 15 min before residual activity measurement. The thermal stability of the purified enzyme was determined at 60°C, pH = 9.0 and 10 for 120 min. All the experiments were carried out in triplicate and the results presented are mean ± standard deviation of the three determinations.

## 3. Results and Discussion

### 3.1. Isolation and Characterization of Xylanase Producing Bacterium

The search for new xylanases with improved thermal and pH stability has led to increased exploitation of microbial sources. In this study, 7 bacterial isolates X1–X7 were isolated from saw-dust dump soil on xylan-agar medium. The isolates were xylanolytic as suggested by clear zones on xylan-agar plates varying from 1.4 to 2.8 cm. The production studies from these isolates suggested xylanase production in the range of 10.4–23.2 IU/mL after 48 hours of incubation (data not shown). The isolate X6 forming biggest clearance zone (2.8 cm) with maximum xylanase production (23.2 ± 0.3 IU/mL) was selected for further studies. No cellulase activity was detected in culture broth (data not shown) indicating that xylanase was free from cellulase contamination.

Morphological and biochemical tests revealed that isolate X6 was rod-shaped, Gram-negative, motile, catalase positive, and oxidase negative. The isolate was moderately thermophilic, capable of growing at 50°C, tolerant to pH up to 10, and could grow in the presence of 8.0% NaCl. However, the optimum temperature and pH for bacterium growth was 35°C and 8.0, respectively. Furthermore, BLAST search of isolate 16S rDNA sequence (763 bp) against nucleotide database showed 99% identity with *Stenotrophomonas maltophilia* strain ATCC 19861(Accession no. NR_040804). The isolate X6 was designated as *S. maltophilia* strain X6 and 16S rRNA gene sequence was deposited in the GenBank (http://www.ncbi.nlm.nih.gov/) under accession number JX220726. While several strains of *S. maltophilia* have been reported earlier for applications involving bioremediation of polycyclic aromatic hydrocarbons [15] and production of alkaline protease [16], its use for production of xylanase has not been described.

### 3.2. Inducible Nature of Xylanase

The replacement of xylan from the production medium with glucose led to absence of clear zone around the well. This suggested that there was no xylanase production when glucose was the carbon source. However, as expected enzyme production was observed in production medium containing xylan as carbon source with a clear zone of 3.3 cm (data not shown). This suggested that the xylanase production from *S. maltophilia* strain X6 was inducible in nature. Most of the microbial xylanases are inducible in nature; however constitutive xylanases have also been reported [17].

### 3.3. Effect of Inoculum Concentration on Production

**Figure 1:**
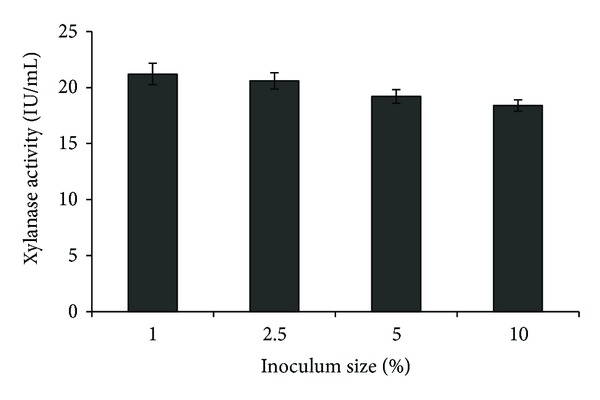
Effect of inoculum size on xylanase production.

The fermentation profile of an organism is affected by the inoculum concentration, as it helps to minimise the time lag in fermentation. It has also been observed that inoculum concentration above 10% is not preferable in industrial processes due to subsequent decline of product yield. Therefore, inoculum concentrations ranging from 1.0% to 10% were studied. The maximum xylanase production of 21.2 ± 1.0 IU/mL was observed at inoculum concentration of 1.0% (Figure 1). The xylanase production studies with *Bacillus mojavensis* AG137 suggested an optimum inoculums concentration of 2.0% [18].

### 3.4. Effect of Carbon and Nitrogen Sources on Production

**Figure 2:**
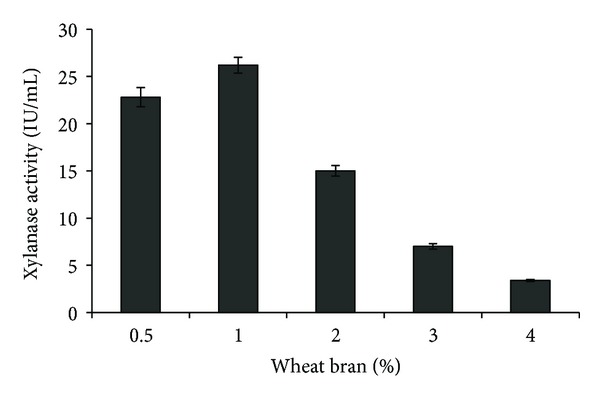
Effect of wheat bran concentration on xylanase production by *S. maltophilia* strain X6.

Xylanase production by microorganisms is regulated by the carbon source in the medium and the induction of xylanase takes place in the presence of inducer molecule [19]. Agroindustrial residues such as wheat bran, corn cobs, wheat straw, rice bran, rice straw, corn stalks, and sugarcane bagasse have been found to support xylanase production [20]. Therefore, different carbon sources like commercial xylans (birchwood and beechwood xylan) and agroindustrial residues were studied for xylanase production by present isolate. Wheat bran supported highest xylanase production (26.4 ± 0.6 IU/mL) followed by birchwood xylan (23.2 ± 0.5 IU/mL) (Table 1). The higher xylanase production in the presence of wheat bran could be due to the presence of significant hemicellulose content. Furthermore, xylanase production (26.2 ± 0.8 IU/mL) was highest at 1.0% wheat bran and then declined gradually with further increase in wheat bran concentrations (Figure 2). This may possibly be due to increased viscosity of medium leading to problems in aeration and nutrient distribution.

The effect of supplementation of additional organic and inorganic nitrogen sources on xylanase production suggested that yeast extract was the best nitrogen source for xylanase production by *S. maltophilia* strain X6 (Table 2). Similar findings had been reported earlier for xylanase production with *B. subtilis* [1]. Compared to organic nitrogen sources, lower yield of xylanase was observed with inorganic nitrogen sources. The organic nitrogen sources such as yeast extract, beef extract, peptone, and tryptone had already been reported for supporting good xylanase production [1].

### 3.5. Effect of Initial Medium pH on Production

The studies with different initial media pH revealed that the best medium pH for xylanase production from present strain was pH = 8.0 with production at pH = 7.0 and pH = 9.0 being almost comparable (Table 3). However, a decline in yield was observed at pH = 6.0 and pH = 10. The results indicated that *S. maltophilia* strain X6 is not only capable of growing well but also producing sufficient xylanase at alkaline pH (8.0-9.0). Similarly, optimum pH for xylanase production by *Bacillus mojavensis* AG 137 was observed to be pH = 8.0 [18].

### 3.6. Purification of Extracellular Xylanase

**Figure 3:**
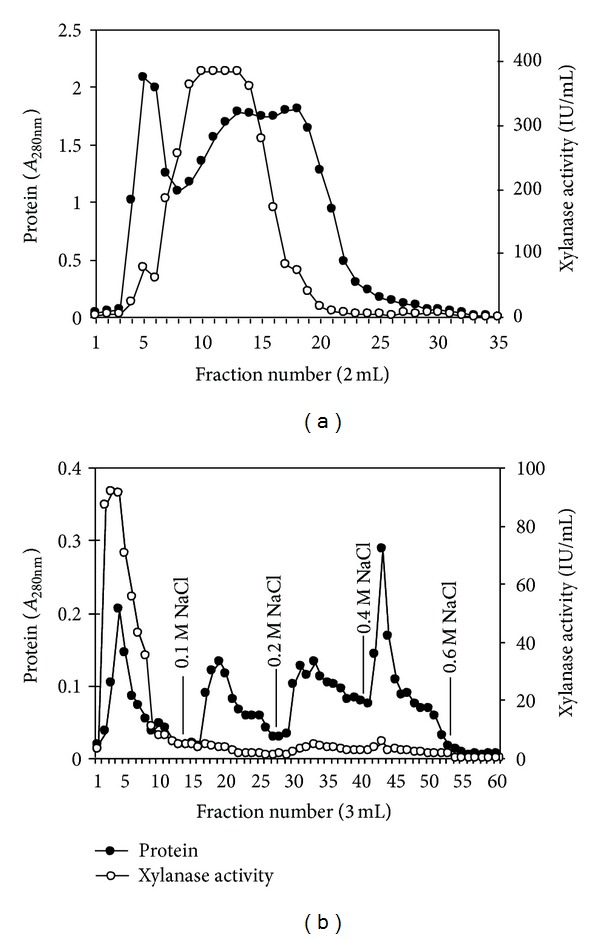
Elution profile of xylanase from *S. maltophilia* on Sephadex G-100 (a) and DEAE-cellulose (b).

Xylanase purification was performed by ammonium sulfate precipitation (0%–80% saturation) followed by Sephadex G-100 and DEAE-cellulose column chromatography (Table 1). The elution profile on Sephadex G-100 showed that xylanase eluted as a broad peak (Figure 3(a)). The fractions number 7–16 having major portion of xylanase were further purified using DEAE-cellulose column, which resulted in the elution of only one protein with xylanase activity in the unbound fractions (Figure 3(b)). The fractions number 2–4 having maximum activity were pooled and used for further study. The final purification fold was 5.43 with a recovery of 19.18% which had specific activity of 313.38 IU/mg protein (Table 4). The recovery of the purified enzyme from *S. maltophilia* strain X6 following three purification steps was higher than reported from *Arthrobacter* sp. with 14% yield using (NH4)_2_SO4 fractionation, Sephadex 200, DEAE-Sepharose FF, and CM-Sepharose FF chromatography [21].

### 3.7. Zymogram Analysis and Molecular Weight Determination

**Figure 4:**
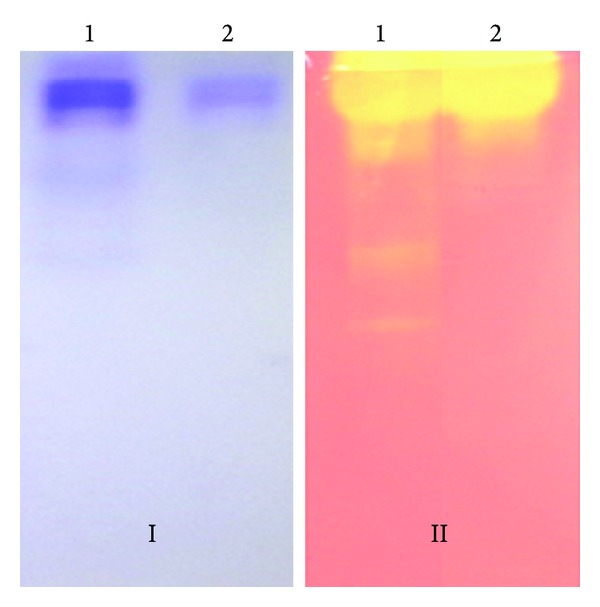
Zymogram analysis of purified *S. maltophilia* strain X6 xylanase. Lane 1: crude xylanase (precipitated by ammonium sulfate); Lane 2: purified xylanase (Sephadex G-100 and DEAE-cellulose chromatography); (I) native PAGE; (II) zymogram.

**Figure 5:**
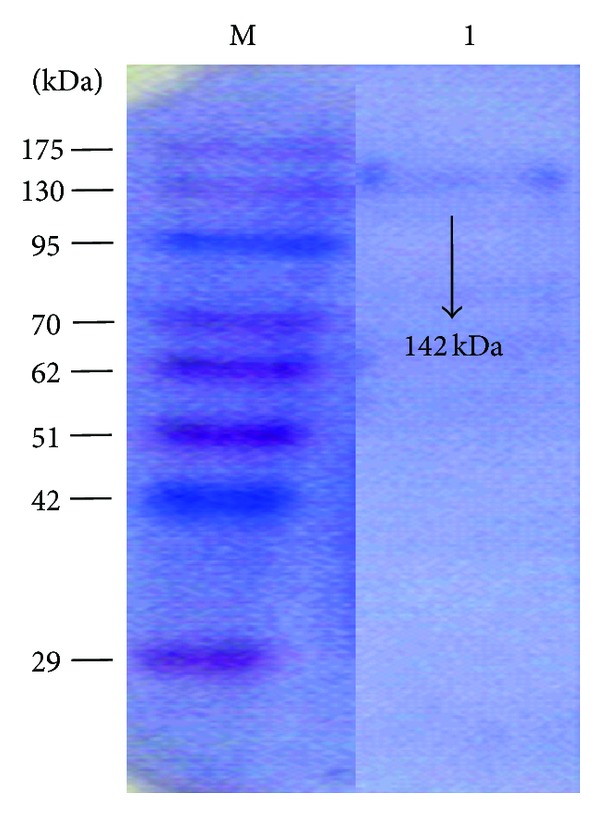
SDS-PAGE analysis of *S. maltophilia* strain X6 xylanase. Lane 1: Sephadex G-100 and DEAE-cellulose purified xylanase; Lane M: protein molecular weight markers.

The zymogram analysis of ammonium sulfate (80%) precipitated sample showed three bands with xylanase activity. However, purified xylanase after Sephadex G-100 and DEAE-cellulose chromatography showed only one band in native PAGE gel as well as in the zymogram (Figure 4). SDS-PAGE analysis of purified xylanase showed a single band ~142 kDa (Figure 5). The molecular weight of isolated xylanase was almost similar to xylanase (xyn 5) from *Aeromonas caviae* W-61 [22]. The molecular weight of xylanases reported from different bacteria varies between 20 and 145 kDa [20, 23]. However, a high molecular weight of 340 kDa xylanase had also been reported from *Bacillus subtilis* [24].

### 3.8. Endolytic Activity of Xylanase

**Figure 6:**
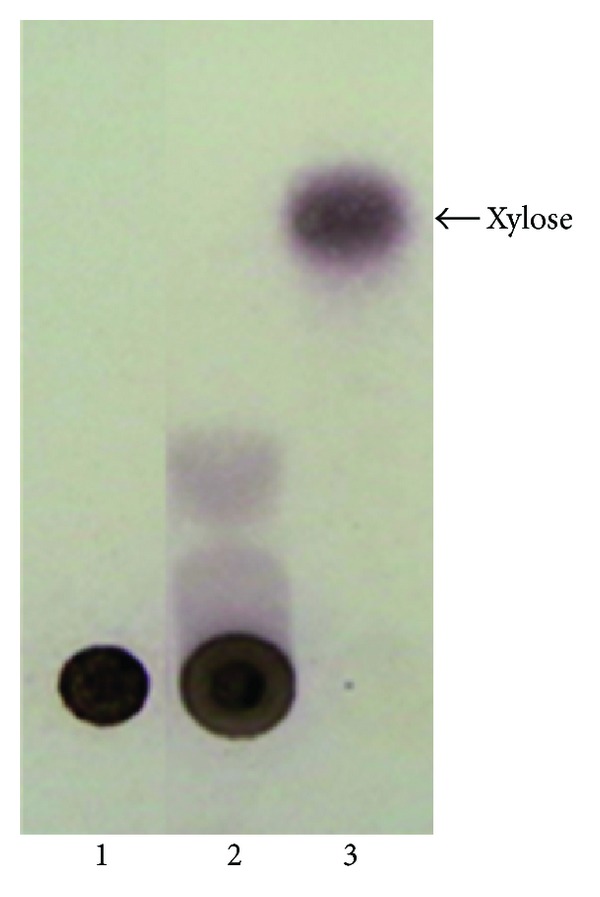
TLC analysis of xylan treated by *S. maltophilia* strain X6 purified xylanase. Lane 1 and lane 3 indicate standard xylan (control) and standard xylose, while the lane 2 represents xylanase-treated xylan showing its degradation products due to action of purified xylanase.

Analysis of standard xylan, xylanase-treated xylan and standard xylose on TLC plate (Figure 6) showed the relative mobility of xylan, xylose, and xylo-oligosaccharides. The xylan being high molecular weight remained at the base while xylose being of the lowest molecular weight moved on the top of TLC plate. The degradation products of xylanase-treated xylan showed mobility between the xylan and xylose. This suggests that the *S. maltophilia* strain X6 xylanase released xylo-oligosaccharides from xylan, which was due to its endoxylanolytic nature. Similarly, xylanase produced by *Bacillus halodurans* S7 was an endoxylanase [25].

### 3.9. pH and Thermal Stability

**Figure 7:**
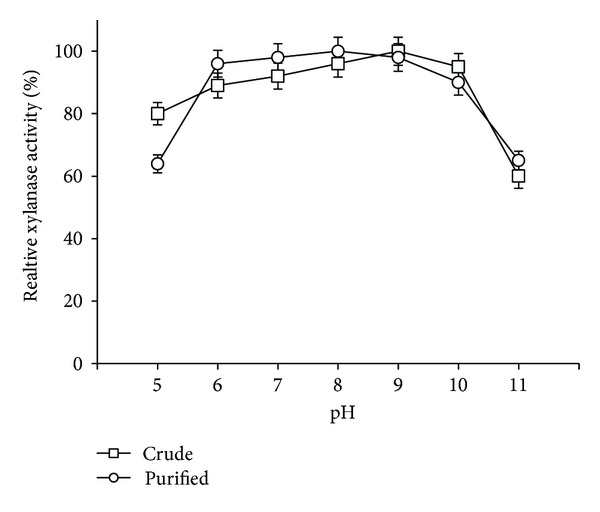
Effect of pH on stability of crude and purified xylanase from *S. maltophilia* strain X6.

The pH stability studies suggested that both crude and purified xylanase from *S. maltophilia* strain X6 were stable (retained >85% activity) in the pH range of pH = 6.0–10.0. Both crude and purified enzyme showed very good stability at pH = 9.0 (>90%) after 30 min preincubation (Figure 7) with significant activity retention (>60%) at pH = 11.0. Although xylanases with optimum activity at pH = 9.0 had been reported earlier; however, significant activity losses were observed at higher pH [26, 27].

**Figure 8:**
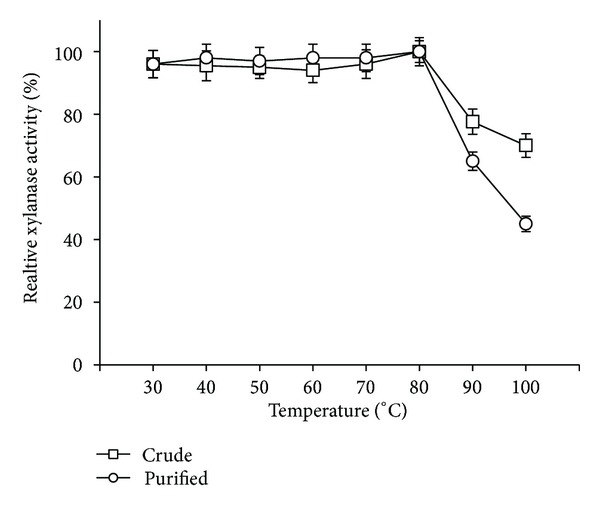
Effect of temperature on stability of crude and purified xylanase of *S. maltophilia* strain X6.

Thermostability studies with crude and purified xylanase at pH = 9.0 showed that enzymes were having good temperature tolerance with residual activity of more than 90%, in the temperature range of 30–80°C and also retained almost 70% and 45% activity at higher temperatures (Figure 8). However, at higher temperature (>80°), the crude xylanase was more stable than the purified one. These results indicated that there may be some other factors in the crude extract that stabilize the xylanase against thermal denaturation. The xylanase from *Geobacillus thermoleovorans* and *Dictyoglomus thermolacticum* showed comparable temperature optima but suffered from lower stability at alkaline pH [28, 29]. Similarly, most of the xylanases reported from *Bacillus* spp. are stable at or below 60°C [20, 27].

**Figure 9:**
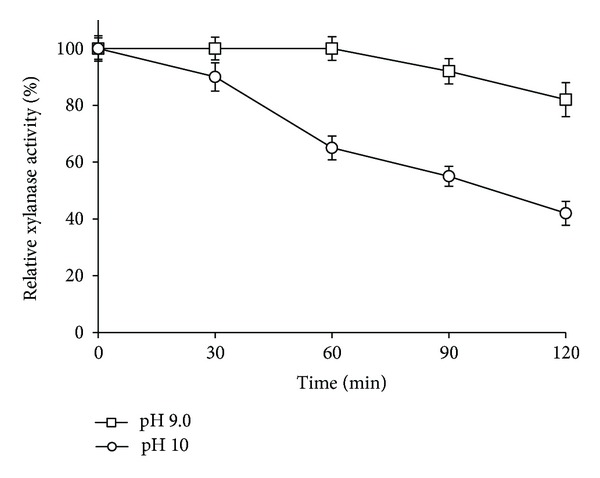
Thermal stability of *S. maltophilia* strain X6 purified xylanase at pH 9 and pH 10 at 60°C. The purified enzyme was incubated in 0.1 M glycine-NaOH for different time intervals and residual activity was determined.

The thermal stability of the purified xylanase was determined at 60 at pH = 9.0 and pH = 10 (Figure 9). At 60°C, the crude xylanase was fully stable after 60 min incubation at pH = 9.0, while it retained 65% of the original activity at pH = 10. It also retained 82 and 42% of its original activity at pH = 9.0 and pH = 10, respectively, after 120 min incubation. The xylanase from an alkaliphilic *Bacillus* sp. V1–4 showed a pH optimum of 6.0–8.5 and a temperature optimum of 55°C [30]. At 60°C and pH = 9.0, this enzyme retained only 15%–20% of its original activity after 30 min incubation.

## 4. Conclusions

The findings of the present study suggest that xylanase from *S. maltophilia* strain X6 was an inducible endoxylanase with good activity retention at 80°C and pH = 9.0 after 30 min of preincubation. Thermostable xylanases from microorganisms are beneficial as they retain their activities at high temperatures and do not require cooling of pretreated biomass. The xylanase produced by present strain may be of potential use in industrial applications such as in biobleaching of pulp and saccharification process. Also, further studies of this enzyme are required to unravel the structural features which are possibly responsible for its thermostability and alkali tolerance.

## Figures and Tables

**Table 1:** Production of xylanase by *S. maltophilia* strain X6 using different carbon sources.

Carbon sources (1% w/v)	Xylanase activity (IU/mL)*
Wheat bran	26.4 ± 0.6
Birchwood xylan	23.2 ± 0.5
Rice bran	20.0 ± 0.4
Beechwood xylan	19.2 ± 0.4

*Results are mean ± SD of three determinations.

**Table 2:** Effect of different nitrogen sources on xylanase production by *S. maltophilia* strain X6.

Nitrogen sources (0.5%)	Xylanase activity (IU/mL)*
Yeast extract	25 ± 0.6
Tryptone	22.8 ± 0.4
Beef extract	20.0 ± 0.6
Peptone	18 ± 0.6
NH_4_NO_3_	10.6 ± 0.4
(NH_4_)_2_SO_4_	9.2 ± 0.4
Urea	8.2 ± 0.4
NaNO_3_	6.8 ± 0.2
KNO_3_	3.6 ± 0.2

*Results are mean ± SD of three determinations.

**Table 3:** Effect of initial pH of the medium on xylanase production by *S. maltophilia* strain X6.

Initial pH of the medium	Xylanase activity (IU/mL)*
6.0	13.8 ± 0.3
7.0	23.2 ± 0.4
8.0	23.8 ± 0.4
9.0	22.8 ± 0.3
10.0	16.4 ± 0.3

*Results are mean ± SD of three determinations.

**Table 4:** Summary of the purification steps of an extracellular xylanase produced by *S. maltophilia* strain X6.

Purification step	Volume (mL)	Total activity (IU)	Total protein (mg)	Specific activity (IU/mg)	Recovery (%)	Purification fold
Culture filtrate	600	13920	241.22	57.71	100	1.00
Ammonium sulfate precipitation	20	7950	96.48	82.40	57.11	1.43
Sephadex G-100	21	5200	36.85	141.11	37.36	2.45
DEAE-cellulose	9	2670	8.52	313.38	19.18	5.43

## References

[1] Sanghi A, Garg N, Sharma J, Kuhar K, Kuhad RC, Gupta VK (2008). Optimization of xylanase production using inexpensive agro-residues by alkalophilic *Bacillus subtilis* ASH in solid-state fermentation. *World Journal of Microbiology and Biotechnology*.

[2] Bajaj BK, Singh NP (2010). Production of xylanase from an alkalitolerant *streptomyces* sp. 7b under solid-state fermentation, its purification, and characterization. *Applied Biochemistry and Biotechnology*.

[3] Kumar KS, Manimaran A, Permaul K, Singh S (2009). Production of *β*-xylanase by a *Thermomyces lanuginosus* MC 134 mutant on corn cobs and its application in biobleaching of bagasse pulp. *Journal of Bioscience and Bioengineering*.

[4] Dhiman SS, Sharma J, Battan B (2008). Industrial applications and future prospects of microbial xylanases: a review. *BioResources*.

[5] Beg QK, Kapoor M, Mahajan L, Hoondal GS (2001). Microbial xylanases and their industrial applications: a review. *Applied Microbiology and Biotechnology*.

[6] Srinivasan MC, Rele MV (1999). Microbial xylanases for paper industry. *Current Science*.

[7] Christopher L, Bissoon S, Singh S, Szendefy J, Szakacs G (2005). Bleach-enhancing abilities of *Thermomyces lanuginosus* xylanases produced by solid state fermentation. *Process Biochemistry*.

[8] Bajaj BK, Sharma M, Sharma S (2011). Alkalistable endo-*β*-1, 4-xylanase production from a newly isolated alkalitolerant *Penicillium* sp. SS1 using agro-residues. *3 Biotech*.

[9] Berg B, von Hofsten B, Pettersson G (1972). Growth and cellulase formation by *Cellvibrio fulvus*. *Journal of Applied Bacteriology*.

[10] Teather RM, Wood PJ (1982). Use of Congo red-polysaccharide interactions in enumeration and characterization of cellulolytic bacteria from the bovine rumen. *Applied and Environmental Microbiology*.

[11] Miller GL (1959). Use of dinitrosalicylic acid reagent for determination of reducing sugar. *Analytical Chemistry*.

[12] Barrow GI, Feltham RKA (1993). *Cowan and Steel’s Manual for the Identification of Medical Bacteria*.

[13] Bradford MM (1976). A rapid and sensitive method for the quantitation of microgram quantities of protein utilizing the principle of protein dye binding. *Analytical Biochemistry*.

[14] Laemmli UK (1970). Cleavage of structural proteins during the assembly of the head of bacteriophage T4. *Nature*.

[15] Juhasz AL, Stanley GA, Britz ML (2000). Microbial degradation and detoxification of high molecular weight polycyclic aromatic hydrocarbons by *Stenotrophomonas maltophilia* strain VUN 10,003. *Letters in Applied Microbiology*.

[16] Kuddus M, Ramteke PW (2011). Production optimization of an extracellular cold active alkaline protease from *Stenotrophomonas maltophilia* MTCC, 7528 and its application in detergent industry. *African Journal of Microbiology Research*.

[17] Bocchini DA, Gomes E, Da Silva R (2008). Xylanase production by *Bacillus circulans* D1 using maltose as carbon source. *Applied Biochemistry and Biotechnology*.

[18] Sepahy AA, Ghazi S, Sepahy MA (2011). Cost-Effective production and optimization of alkaline xylanase by indigenous *Bacillus mojavensis* AG137 fermented on agricultural waste. *Enzyme Research*.

[19] Parachin NS, Siqueira S, de Faria FP, Torres FAG, de Moraes LMP (2009). Xylanases from *Cryptococcus flavus* isolate I-11: enzymatic profile, isolation and heterologous expression of CfXYN1 in *Saccharomyces cerevisiae*. *Journal of Molecular Catalysis B*.

[20] Kulkarni N, Shendye A, Rao M (1999). Molecular and biotechnological aspects of xylanases. *FEMS Microbiology Reviews*.

[21] Khandeparkar RDS, Bhosle NB (2006). Isolation, purification and characterization of the xylanase produced by *Arthrobacter* sp. MTCC 5214 when grown in solid-state fermentation. *Enzyme and Microbial Technology*.

[22] Roy N, Kamio Y (2002). Purification, characterization and amplification of a 1.8 kbp fragment of xylanase 5 from *Aeromonas caviae* W-61. *Indian Journal of Biochemistry and Biophysics*.

[23] Subramaniyan S, Prema P (2002). Biotechnology of microbial xylanases: enzymology, molecular biology, and application. *Critical Reviews in Biotechnology*.

[24] Sá-Pereira P, Costa-Ferreira M, Aires-Barros MR (2002). Enzymatic properties of a neutral endo-1,3(4)-*β*-xylanase Xyl II from *Bacillus subtilis*. *Journal of Biotechnology*.

[25] Mamo G, Hatti-Kaul R, Mattiasson B (2006). A thermostable alkaline active endo-*β*-1-4-xylanase from *Bacillus halodurans* S7: purification and characterization. *Enzyme and Microbial Technology*.

[26] Nakamura S, Wakabayashi K, Nakai R, Aono R, Horikoshi K (1993). Purification and some properties of an alkaline xylanase from alkaliphilic *Bacillus* sp. strain 41M-1. *Applied and Environmental Microbiology*.

[27] Gessesse A, Gashe BA (1997). Production of alkaline xylanase by an alkaliphilic *Bacillus* sp. isolated from an alkaline soda lake. *Journal of Applied Microbiology*.

[28] Sharma A, Adhikari S, Satyanarayana T (2007). Alkali-thermostable and cellulase-free xylanase production by an extreme thermophile *Geobacillus thermoleovorans*. *World Journal of Microbiology and Biotechnology*.

[29] Mathrani IM, Ahring BK (1992). Thermophilic and alkalophilic xylanases from several *Dictyoglomus* isolates. *Applied Microbiology and Biotechnology*.

[30] Yang VW, Zhuang Z, Elegir G, Jeffries TW (1995). Alkaline-active xylanase produced by an alkaliphilic *Bacillus* sp. isolated from kraft pulp. *Journal of Industrial Microbiology*.

